# Optical Coherence Tomography Angiography in Patients with Mixed Connective Tissue Disease

**DOI:** 10.3390/biomedicines14030612

**Published:** 2026-03-09

**Authors:** Magdalena Szeretucha, Katarzyna Paczwa, Katarzyna Romanowska-Próchnicka, Sylwia Ornowska, Radosław Różycki, Joanna Gołębiewska

**Affiliations:** 1 Department of Ophthalmology, Military Institute of Aviation Medicine, 01-755 Warsaw, Polandjoanna.golebiewska@wp.pl (J.G.); 2 Department of Biophysics, Physiology and Pathophysiology, Medical University of Warsaw, 02-004 Warsaw, Poland; 3 Clinic and Polyclinic of Systemic Connective Tissue Disease, National Institute of Geriatrics, Rheumatology and Rehabilitation, 02-637 Warsaw, Poland

**Keywords:** mixed connective tissue disease, retinal microvasculature, retinal vessel density, optical coherence tomography angiography

## Abstract

**Background**: Mixed connective tissue disease (MCTD) is a rare systemic autoimmune disease which presents with clinical features that overlap with at least two connective tissue disorders, including systemic lupus erythematosus (SLE), systemic sclerosis (SSc), polymyositis (PM), dermatomyositis (DM), and rheumatoid arthritis (RA). It is characterized by the presence of anti-ribonucleoprotein (anti-U1RNP) antibodies. The mechanism of the vasculopathy associated with MCTD remains largely unknown. Optical coherence tomography angiography (OCTA) is a non-invasive imaging method of the microvasculature of the retina and choroid, providing the assessment of retinal perfusion. **Objectives**: The aim of the study was to evaluate the optical coherence tomography angiography (OCTA) parameters in patients with mixed connective tissue disease compared to healthy individuals. **Methods**: In this study, we compared the following parameters between patients with MCTD and healthy subjects: foveal avascular zone (FAZ), FAZ perimeter (PERIM), flow density (FD), choriocapillaris flow area (CCFA), outer retina flow area (ORFA), and foveal and parafoveal mean superficial and deep vessel density. **Results**: Parafoveal mean superficial vessel density and parafoveal mean deep vessel density were significantly lower in the MCTD group than in controls. The FAZ, FAZ PERIM, and FD values in the patients with MCTD were lower than in the control group and statistically significant for all parameters. **Conclusions**: The present study’s findings suggest the presence of ocular vascular abnormalities in patients suffering from MCTD. These abnormalities are characterized by decreased retinal vessel density and lower choriocapillaris flow. The results of the study demonstrate the significant role of OCTA in the diagnosis and monitoring of microvascular changes in patients with MCTD.

## 1. Introduction

Mixed connective tissue disease (MCTD) is a rare systemic autoimmune disorder, characterized by the clinical features of at least two connective tissue diseases such as systemic lupus erythematosus (SLE), systemic sclerosis (SSc), polymyositis (PM), dermatomyositis (DM), and rheumatoid arthritis (RA) [1,2]. The disease is also associated with high titters of anti-ribonucleoprotein antibodies (anti-U1-RNP) [1,3]. Despite the fact that the pathophysiology of MCTD remains largely unknown, it is understood to be multifactorial in nature. Currently, there are no universally agreed diagnostic criteria for MCTD [1,4].

The pathogenesis of MCTD is characterized by excessive production of pro-inflammatory cytokines, such as IL-1 and IL-6, which contribute significantly to inflammation and tissue damage. Additionally, the disease mechanism involves the direct action of anti-U1RNP antibodies on endothelial cells, leading to the production of adhesion molecules like ICAM-1 and ELAM-1. These molecules facilitate the adhesion and recruitment of inflammatory cells to the vascular wall, resulting in endothelial damage and contributing to the development of vascular complications. This process ultimately leads to vascular dysfunction and further exacerbates inflammation and tissue damage [1,4,5].

The clinical manifestations and severity of MCTD can vary significantly among individuals. However, the most frequent symptoms include Raynaud’s phenomenon (RP), digital edema (puffy fingers), arthralgia, and muscle weakness. In more severe cases, patients may develop interstitial lung disease (ILD), and less frequently, pulmonary arterial hypertension (PAH) [6]. Vascular pathologies can affect different organs, including the eyes.

Optical coherence tomography angiography (OCTA) is a non-invasive imaging technique that generates detailed, three-dimensional images of the retinal and choroidal microvasculature. This enables the assessment of retinal blood flow, obviating the need for contrast dye [7,8].

The aim of the study was to evaluate the optical coherence tomography angiography (OCTA) parameters in patients with mixed connective tissue disease compared to healthy individuals.

## 2. Materials and Methods

This cross-sectional study was conducted between November 2022 and November 2023 at the Department of Ophthalmology, Military Institute of Aviation Medicine in Warsaw, Poland. This study received ethical approval from the National Institute of Geriatrics, Rheumatology, and Rehabilitation Ethics Committee (KBT-6/5/2022). All study procedures were conducted in accordance with the Declaration of Helsinki. Following a comprehensive explanation of the study’s nature, informed consent was obtained from all subjects involved in the study.

The study group consisted of patients diagnosed with mixed connective tissue disease, aged between 18 and 64 years, from the Department of Connective Tissue Diseases, National Institute of Geriatrics, Rheumatology, and Rehabilitation in Warsaw, Poland, and healthy controls recruited during standard follow-up visits to the Department of Ophthalmology at the Military Institute of Aviation Medicine. Patients were excluded if they had refractive errors such as myopia (≤−3.0 D) or hyperopia (≥+3.0 D), or if they had eye diseases such as glaucoma, retinal or choroidal pathology, or uveitis. Further exclusion criteria included a signal quality below 6 and a lack of written consent to participate in the study.

A complete ophthalmological examination was performed for each participant. The examination included the following procedures: refraction testing, assessment of best-corrected visual acuity (BCVA), tonometry, slit-lamp biomicroscopy of the anterior segment, and dilated fundus examination using a 90-diopter lens. BCVA was measured monocularly using LogMAR charts (Lighthouse International, New York, NY, USA) at a distance of 5 m. Visual acuity was generally preserved in both groups (range: 0.8–1.0 in the MCTD group and 0.9–1.0 in the control group). The slight reductions in BCVA observed in the study group were mostly due to tear film disorder or an early-stage cataract (either age-related or associated with chronic corticosteroid therapy), rather than retinal pathology.

**Figure 1:**
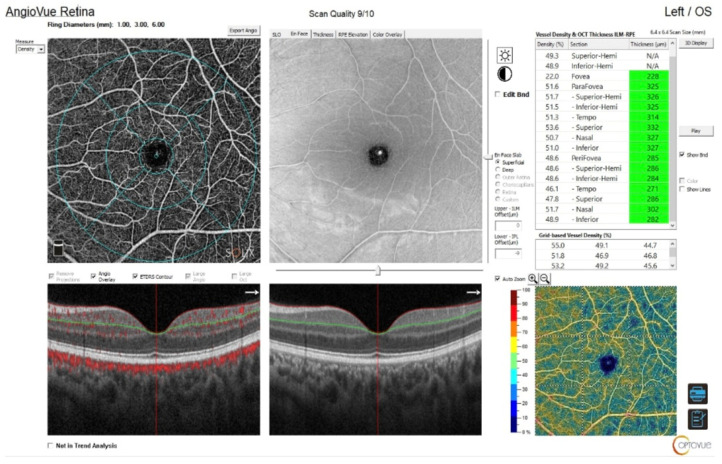
Representative OCTA vessel density report of the patient with MCTD. The figure shows the image of the macular vessel in the superficial plexus calculated separately in five regions (fovea, temporal, superior, nasal, and inferior) based on the ETDRS contour, OCT En face image, B-scans, and the results of quantitative analysis by the software.

OCTA of the retina was performed using the SOLIX™ (version 1.x) with the AngioVue^®^ software feature (Optovue, Inc., Freemont, CA 94538, USA). The scanning area was captured in 6.4 × 6.4 mm^2^ sections, automatically centered on the fovea. Macular outcomes were measured focusing on the fovea (0–1 mm) and parafoveal regions. The parafoveal area was subdivided into quadrants: temporal, superior, nasal, and inferior. The parafoveal ring was delineated, with inner and outer ring diameters of 1 and 3 mm, respectively (Figure 1).

**Figure 2:**
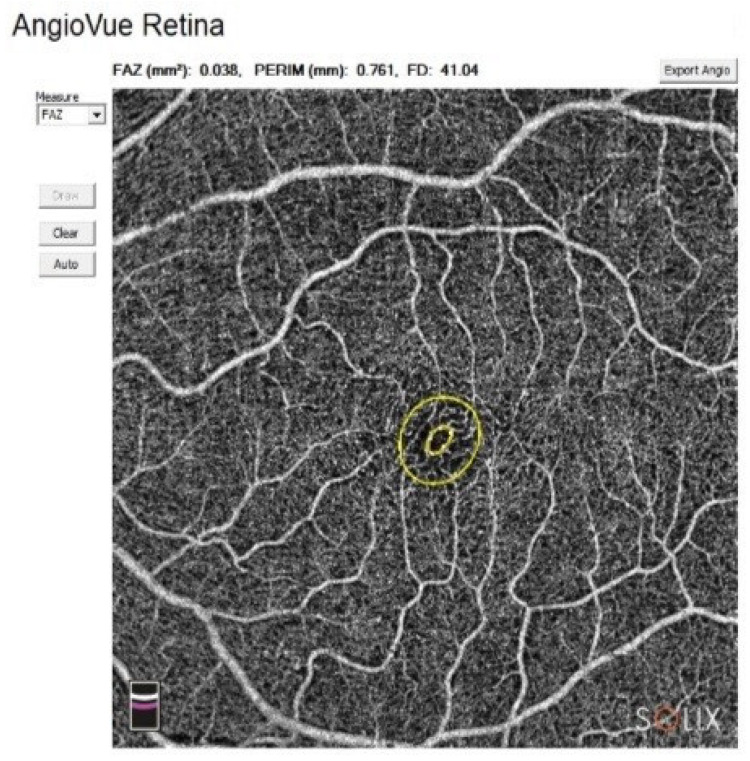
Representative FAZ report of the patient with MCTD. The figure shows an example of an OCTA scan centered on the fovea. The FAZ is shown as the marked zone.

The AngioAnalytics™ software feature of AngioVue^®^ is indicated for the measurement of vascular density, the foveal avascular zone, and flow area. The system measures the vessel density of the selected plexus and displays it in percentage. For the superficial vascular layer, the slab extends from the ILM to 10 μm above the IPL. For the deep vascular layer, the slab extends from 10 μm above the IPL to 10 μm below the OPL. The measurement of flow area is based on AngioVue^®^ retina scans, which detect flow in the outer retina slab (OPL + 10 µm to BRM − µm) and the choroidal capillaries slab (BRM − 10 µm to BRM + 30 µm). The FAZ was evaluated by measuring the following: area (mm^2^), perimeter (mm), and FD—vessel density of the 300 µm width ring surrounding the FAZ (%). The FAZ was measured using the automated boundary detection provided by the AngioVue^®^ software (Figure 2).

For each eye, two scans were obtained, and the one with the highest image quality was selected for analysis.

Numerical traits were described by their mean, standard deviation, median, and lower-to-upper quartile values. Categorical variables were presented as integers and percentages.

Normality of distribution was tested by using the Shapiro–Wilk W test. Levene’s test was used to assess the homogeneity of variances. A multifactor analysis of variance (ANOVA) was performed for normally distributed numerical traits with homogenic variances in order to estimate the significance of differences in the investigated traits by prevalence of MCTD. Generalized linear models were fitted when dealing with non-normally distributed variables or heterogenic variances.

Due to the fact that the multifactorial analyses included measurements from two eyes per patient, a standard error correction consisting of intra-subject correlations was applied.

A level of *p* < 0.05 was considered statistically significant. All the statistical procedures were performed using Statistica 13.3 (TIBCO Software Inc., Palo Alto, CA, USA).

## 3. Results

The present study comprised a total of 83 individuals, of whom 71 were female (85.54%), and 12 were male (14.46%). The study population included 51 eyes from 27 participants with mixed connective tissue disease (32.53%), which were compared with OCTA measurements from 112 eyes of 56 healthy control participants (67.47%). The demographic structure of the study cohort was found to be balanced. The median age was recorded as 44 years (quartiles 34–51 years) (Table 1).

The superficial vessel density (SVD) in the study participants with MCTD was found to be comparable to that of the comparison group, exhibiting no statistically significant difference in the fovea (*p* = 0.1501), with respective values of 28.82% (SD 5.83%) and 27.62% (SD 5.48%). However, statistically significant variations were identified in the temporal (*p* = 0.0408) and superior (*p* = 0.0004) quadrants, with respective values of 48.22% (SD 3.21%) versus 49.14% (SD 2.35%) and 48.87% (SD 3.21%) versus 50.42% (SD 2.23%) (Table 2).

The deep vessel density (DVD) measurements demonstrated that the deep vessels in the fovea are statistically significantly thicker in the affected group than in the control group, with a mean of 31.23% (SD 6.36%) versus 28.83% (SD 5.55%) (*p* = 0.0101). However, the mean DVD was significantly lower in individuals with MCTD diagnosed, measuring 53.15% (SD 3.51%) compared to 54.34% (SD 1.25%) (*p* = 0.0014). Impairments of the DVD in the affected subjects were also significantly pronounced in the temporal (*p* = 0.0252), superior (*p* = 0.0100), and nasal (*p* < 0.0001) quadrants. In the temporal region, the proportion of subjects exhibiting impairment was 53.33% (SD 3.78%) compared to 54.32% (SD 1.84%), while in the superior region, the proportion was 53.16% (SD 4.26%) versus 54.37% (SD 1.83%), and nasal 52.34% (SD 4.46%) versus 54.57% (SD 1.86%) (Table 3).

The mean FAZ area of the healthy control group was 0.2858 mm^2^ (SD 0.1357 mm^2^), whereas in the MCTD group, it was 0.2356 mm^2^ (SD 0.1184 mm^2^), which was a statistically significant difference (*p* = 0.0191). A difference in FAZ perimeter, statistically confirmed, was observed between the control group (2.05 mm, SD 0.50 mm) and the MCTD group (1.85 mm, SD 0.50 mm). Finally, the FD value in the control group was 51.53% (SD 4.79%) compared with 49.40% (SD 4.96%) in the MCTD group, which was a statistically significant difference (*p* = 0.0089) (Table 4).

With regard to the flow areas, equalization was observed between the study groups in the outer retina (*p* = 0.8342). However, a statistically significant difference in the flow area of the choriocapillaris was identified between groups in the MCTD subjects, with a mean of 1.91 mm^2^ (SD 0.43 mm^2^) versus 2.04 mm^2^ (SD 0.29 mm^2^) (*p* = 0.0433) (Table 5).

## 4. Discussion

Retinal vasculitis has been reported in a small number of cases in patients with MCTD, but the main mechanism underlying the vasculopathy remains undefined. A search of the PubMed database revealed a limited number of case reports describing retinal vascular involvement [9,10], and the discussion of this study is therefore based on those of related studies in other diseases, including SLE, SSc, PM, DM, and RA.

The examination of the retinal microvasculature was performed using OCTA in participants diagnosed with MCTD, as well as in a control group of healthy individuals.

Endothelial injury plays a main role in the pathogenesis of systemic sclerosis (SSc), serving as a unifying factor that links the key processes of vasculopathy, chronic inflammation, and fibrosis. Given that vasculopathy is a hallmark of this disease, it is plausible to hypothesize that alterations in retinal vasculature may also occur, reflecting the underlying vascular pathogenesis of SSc [9,10].

In their study, Kok et al. revealed a significantly lower vessel density in both the superficial and deep capillary plexuses in patients with SSc compared to a control group, while FAZ and FAZ perimeter were significantly reduced in these patients. A significant decrease in choroidal capillary flow was noted in patients with SSc, and their findings were similar to those reported in this study [11].

As demonstrated by Rothe et al., macular vessel density in the superficial capillary plexus was lower in individuals with SSc than in healthy controls [12].

Mihailovic et al., in their study comparing patients with SSc to healthy controls, found that vessel density in the superficial capillary plexus and choriocapillaris was lower in patients with SSc. The FAZ did not demonstrate a statistically significant difference between the two groups (*p* = 0.474) [13]. These results are similar to those of the study by Paczwa K. et al. [8].

Rommel et al. reported a significant reduction in the perfusion of the superficial and deep retinal plexuses in patients with SSc [14].

Similarly, Foti et al. reported a significant decrease in vessel density across the entire superficial and deep capillary plexuses within the ETDRS grid in patients compared with healthy controls. The observed reduction in retinal vascular density, together with the enlargement of the FAZ, may result from capillary loss and vascular fibrosis. These findings support the hypothesis of diffuse microvascular dysfunction in systemic sclerosis [15].

Wang X et al. studied retinal vascular alterations in patients with SLE using OCTA. Compared with healthy controls, patients with SLE exhibited a significant reduction in vessel density in both the superficial and deep retinal plexuses. Despite this reduction in vessel density, no significant difference in FAZ was observed between the study group and the healthy control group [16].

Ferreira A et al. reported results that were comparable to those of the above authors. In both the superficial and deep capillary plexuses, patients with SLE exhibited significantly reduced vessel density and FD, with all *p* values < 0.005. However, no significant differences in FAZ values were observed. These findings on OCTA provide valuable insight into the early vascular changes associated with SLE and may serve as quantitative indicators for disease monitoring [17].

In a similar manner to the previous study, Pelegrin L et al. wanted to detect early ocular changes in patients with SLE using OCTA. The perifoveal microvasculature perfusion parameters in the eyes of patients with SLE were assessed in comparison with those observed in a healthy group. The results obtained revealed a significant decrease in the perifoveal capillary network of patients with SLE when compared with the control group. Furthermore, a positive correlation was identified between the duration of the disease and the extent of the decrease in OCTA parameters, with longer disease duration (>10 years) being associated with further decreases in OCTA parameters. The FAZ area did not differ between patients with SLE and controls, or among patients with SLE with different disease durations [18].

In their study, Huang BZ et al. revealed that superficial vessel density in patients with DM was significantly lower in the regions assessed compared with the control group (*p* < 0.001). The authors of the study predict that changes in SVD may serve as an important indicator for assessing the progression of DM. The use of the OCTA technology facilitates the assessment of vessel density and thus the severity of occlusion. The utilization of OCTA in the early diagnosis and observation of patients should be carried out to monitor ischaemia leading to irreversible vision loss [19].

In this discussion, the results were compared with those of three other studies conducted among patients with RA. The first study by Ayar K et al. showed that the density of the retinal capillary plexus in the macula (SCP and DCP blood supply regions) was lower in patients with RA than in healthy controls. The assessment parameters of the FAZ, with the exception of FD, were found to be similar between patients with RA and healthy controls. The mean FD in patients with RA was lower, which was a statistically significant difference (*p* = 0.003) [20]. In the second study, Hsuan-Yi Lee et al. observed significantly reduced vessel density in the superficial and deep layers of the retina in the RA group compared with the control group (*p* < 0.05) [21]. In the third study, OCTA data analysis by Iacono P et al. showed a statistically significant reduction in vessel density, especially in the superior quadrant of the superficial retinal capillary plexus, in patients with RA, indicating results that are similar to those of our investigation. No statistically significant difference was observed between the two groups with respect to the FAZ value, although a slight decrease was noted in the RA group [22]. These findings indicate early microvascular changes in patients with RA, which may occur before clinical symptoms become apparent.

Although a larger FAZ is theoretically expected in patients with microangiopathy, there are several factors that could explain why a larger FAZ was found in the control group. Firstly, microvascular involvement in MCTD can be heterogeneous and stage-dependent. In the early stages or during the predominantly inflammatory phase, endothelial activation and capillary dilation may predominate over capillary dropout. This may result in relatively smaller or even preserved FAZ measurements. Secondly, microvascular remodeling or capillary recruitment in response to chronic, mild ischemia may partially compensate for capillary rarefaction at the foveal border. Furthermore, methodological factors inherent to OCT-A assessment, such as signal strength, segmentation algorithms, scan size, and FAZ delineation method (manual or automatic), may influence absolute FAZ values, particularly in relatively small study groups, as was the case in our study.

A survey of the literature revealed the absence of studies that have systematically evaluated OCTA findings in patients with MCTD, although there are studies using OCT in patients with SLE, SSc, PM, DM, and RA. This study’s strengths include the detailed patient selection, taking into consideration parameters that could confound the interpretation of the results, such as patient age, refractive error, axial length, and visual acuity. The use of automatic measurements in this study eliminates the potential errors associated with manual methods. The present study is not without its limitations. Firstly, the findings, especially in a small sample such as the one in this study, need to be verified in future studies. It is also important to take into account other factors that may affect vessel density. However, the present study is the first to reveal significant changes in the retinal circulation in patients with MCTD, which were consistent with the changes previously described in individual disease entities whose clinical manifestations occur in patients with MCTD.

## 5. Conclusions

The present study demonstrated retinal vascular changes in patients with mixed connective tissue disease. OCTA proved to be an effective method for evaluating microvascular impairment in patients with MCTD. The findings highlight the importance of routine ophthalmic examination for early identification and management of ocular manifestations in these patients. Nonetheless, additional research involving a larger patient cohort is necessary to corroborate these results.

## Figures and Tables

**Table 1:** Baseline characteristics of the study cohort (*discrete variables*) (n = 83 individuals).

Analyzed Trait *	Overall	MCTD	Controls	*Ip*-Value
**No. of participants, *n* (%)**	83	27 (32.53)	56 (67.47)	
**No. of eyes, *n* (%)**	163	51 (31.29)	112 (68.71)	
**Gender, *n* (%)**				
− **Female**	71 (85.54)	24 (88.89)	47 (83.93)	=0.5496
− **Male**	12 (14.46)	3 (11.11)	9 (16.07)	
Age (years), *Me* (*Q*_1_*–Q*_3_) **	44 (34–51)	44 (34–51)	38 (31–54)	=0.4043

* Statistical measures used: n—number; %—percentage; Me—median; Q—quartiles. ** Controlled for gender.

**Table 2:** Descriptive statistics for the superficial vessel density (%) as measured in the study group according to the prevalence of MCTD (n = 163 eyes).

Superficial Vessel Density (%)	Prevalence of MCTD	Statistical Parameter				*p*-Value *
		*M*	*SD*	*Me*	*Q*_1_ *–Q*_3_	
**Foveal**	**MCTD**	28.82	5.83	28.60	24.40–33.00	=0.1501
	**Controls**	27.62	5.48	27.45	23.90–31.40	
**Parafoveal, temporal**	**MCTD**	**48.22**	**3.21**	**48.85**	**47.30–50.20**	**=0.0408**
	**Controls**	**49.14**	**2.35**	**49.65**	**48.10–50.85**	
**Parafoveal, superior**	**MCTD**	**48.87**	**3.21**	**49.45**	**48.10–51.00**	**=0.0004**
	**Controls**	**50.42**	**2.23**	**50.75**	**49.40–51.80**	
**Parafoveal, nasal**	**MCTD**	47.95	4.09	49.10	46.70–50.40	=0.1035
	**Controls**	48.87	2.95	49.10	48.05–50.70	
**Parafoveal, inferior**	**MCTD**	49.59	2.93	50.30	47.80–51.70	=0.1998
	**Controls**	50.16	2.50	50.55	48.80–51.90	
**Parafoveal, Mean SVD**	**MCTD**	48.66	2.99	49.06	47.30–50.70	**=0.0173**
	**Controls**	49.65	2.18	50.10	48.76–51.21	

* Controlled for the study subjects’ age and gender.

**Table 3:** Descriptive statistics for the deep vessel density (%) as measured in the study group according to the prevalence of MCTD (n = 163 eyes).

Deep Vessel Density (%)	Prevalence of MCTD	Statistical Parameter				*p*-Value *
		*M*	*SD*	*Me*	*Q*_1_ *–Q*_3_	
**Foveal**	**MCTD**	**31.23**	**6.36**	**32.75**	**25.80–36.40**	**=0.0101**
	**Controls**	**28.83**	**5.55**	**29.75**	**25.20–33.00**	
**Parafoveal, temporal**	**MCTD**	**53.33**	**3.78**	**54.05**	**52.10–55.70**	**=0.0252**
	**Controls**	**54.32**	**1.84**	**54.40**	**53.05–55.55**	
**Parafoveal, superior**	**MCTD**	**53.16**	**4.26**	**54.60**	**52.10–55.70**	**=0.0100**
	**Controls**	**54.37**	**1.83**	**54.55**	**53.25–55.85**	
**Parafoveal, nasal**	**MCTD**	**52.34**	**4.46**	**52.95**	**51.50–55.00**	**<0.0001**
	**Controls**	**54.57**	**1.86**	**54.80**	**53.50–55.95**	
**Parafoveal, inferior**	**MCTD**	53.59	3.42	54.00	53.00–55.40	=0.2366
	**Controls**	54.11	1.80	54.30	52.80–55.45	
**Parafoveal, Mean DVD**	**MCTD**	**53.15**	**3.51**	**54.11**	**52.57–54.83**	**=0.0014**
	**Controls**	**54.34**	**1.25**	**54.45**	**53.52–55.35**	

* Controlled for the study subjects’ age and gender.

**Table 4:** Descriptive statistics for the foveal avascular zone (µm) as measured in the study group according to the prevalence of MCTD (n = 163 eyes).

Analyzed Trait	Prevalence of MCTD	Statistical Parameter				*p*-Value *
		*M*	*SD*	*Me*	*Q*_1_ *–Q*_3_	
**FAZ** **(mm^2^)**	**MCTD**	**0.2356**	**0.1184**	**0.1985**	**0.1530–0.3240**	**=0.0191**
	**Controls**	**0.2858**	**0.1357**	**0.2720**	**0.2060–0.3410**	
**PERIM** **(mm)**	**MCTD**	**1.85**	**0.50**	**1.74**	**1.50–2.23**	**=0.0160**
	**Controls**	**2.05**	**0.50**	**2.06**	**1.77–2.29**	
**FD-300** **(%)**	**MCTD**	**49.40**	**4.96**	**49.64**	**47.27–53.20**	**=0.0089**
	**Controls**	**51.53**	**4.79**	**52.44**	**49.50–54.17**	

* Controlled for the study subjects’ age and gender.

**Table 5:** Descriptive statistics for the flow areas (mm^2^) as measured in the study group according to the prevalence of MCTD (n = 163 eyes).

Flow Area (mm^2^)	Prevalence of MCTD	Statistical Parameter				*p*-Value *
		*M*	*SD*	*Me*	*Q*_1_ *–Q*_3_	
**Outer retina**	**MCTD**	1.12	0.35	1.10	0.97–1.23	=0.8342
	**Controls**	1.13	0.25	1.05	0.97–1.20	
**Choriocapillaris**	**MCTD**	**1.91**	**0.43**	**1.92**	**1.80–2.16**	**=0.0433**
	**Controls**	**2.04**	**0.29**	**2.04**	**1.83–2.25**	

* Controlled for the study subjects’ age and gender.

## Data Availability

The data presented in this study are available on request from the corresponding author.

## Abbreviations

The following abbreviations are used in this manuscript:

MCTD	Mixed Connective Tissue Disease
SLE	Systemic Lupus Erythematosus
SSc	Systemic Sclerosis
PM	Polymyositis
DM	Dermatomyositis
RA	Rheumatoid Arthritis
anti-U1RNP	Anti-ribonucleoprotein
OCTA	Optical Coherence Tomography Angiography
FAZ	Foveal Avascular Zone
FD	Flow Density
CCFA	Choriocapillaris Flow Area
ORFA	Outer Retina Flow Area
PAH	Pulmonary Arterial Hypertension
RP	Raynaud’s Phenomenon
ILD	Interstitial Lung Disease
ILM	Internal Limiting Membrane
IPL	Inner Plexiform Layer
BRM	Bruch’s membrane
SD	Standard Deviation
SVD	Superficial Vessel Density
DVD	Deep Vessel Density
BCVA	Best Corrected Visual Acuity
M	Mean
Me	Median
Q	Quartiles
